# A Critical Role for Cysteine 57 in the Biological Functions of Selenium Binding Protein-1

**DOI:** 10.3390/ijms161126043

**Published:** 2015-11-18

**Authors:** Qi Ying, Emmanuel Ansong, Alan M. Diamond, Wancai Yang

**Affiliations:** 1 Department of Pathology, Xinxiang Medical University, Xinxiang 453003, China; yingq1@hotmail.com; 2 Department of Pathology, University of Illinois at Chicago, Chicago, IL 60612, USA; eanson2@uic.edu; 3 College of Food Science and Technology, Nanjing Agricultural University, Nanjing 210095, China

**Keywords:** selenium-binding protein1, glutathione peroxidase 1, selenite cytotoxicity, mitochondrial damage

## Abstract

The concentration of selenium-binding protein1 (SBP1) is often lower in tumors than in the corresponding tissue and lower levels have been associated with poor clinical outcomes. SBP1 binds tightly selenium although what role selenium plays in its biological functions remains unknown. Previous studies indicated that cysteine 57 is the most likely candidate amino acid for selenium binding. In order to investigate the role of cysteine 57 in SBP1, this amino acid was altered to a glycine and the mutated protein was expressed in human cancer cells. The SBP1 half-life, as well as the cellular response to selenite cytotoxicity, was altered by this change. The ectopic expression of SBP1^GLY^ also caused mitochondrial damage in HCT116 cells. Taken together, these results indicated that cysteine 57 is a critical determinant of SBP1 function and may play a significant role in mitochondrial function.

## 1. Introduction

The human selenium binding protein-1 (SBP1, SELENBP1) is ubiquitously expressed in a variety of tissues including brain, liver, heart, lung, and kidney [1,2,3,4,5]. Levels of SBP1 have often been found to be lower in tumors as compared to the corresponding tissues and lower levels are often associated with poor clinical outcome [6,7,8,9,10]. In addition, SBP1 has been reported to interact with hypoxia-inducible factor-1 α (HIF1α) and von Hippel-Lindau protein (pVHL)-interacting deubiquitinating enzyme 1 (VDU1), indicating that SBP1 may participate in antioxidant responses and/or protein degradation [3,11]. SBP1 may also be involved in intra-Golgi transport and selenium metabolism [12,13]. However, the specific biological functions of SBP1 and its putative role in cancer etiology remain to be resolved.

Previously, we have reported a physical interaction between SBP1 and another selenium-containing protein, glutathione peroxidase 1 (GPx1) that contains selenium in the form of selenocysteine [5]. Whether this interaction between SBP1 and GPx1 requires the selenocysteine in GPx1 or the selenium moiety of SBP1 is not known. A recent study investigating the nature of the association between selenium and SBP1 indicated that cysteine 57 was the likely selenium binding site because it is the only freely-accessible cysteine exposed to solvent and surrounded by hydrophobic residues [4]. However, an *in vitro* study of *Arabidopsis thaliana* SBP1 using purified recombinant protein indicated that cysteines 21 and 22 could bind a single atom of selenium to form a R-S-Se (II)-S-R-type complex [12]. Based on the homology of *Arabidopsis thaliana* and human SBP1, it was predicated that cysteines 5 and 8 were possible candidates for selenium binding in the human SBP1 protein. In order to examine the importance of cysteine 57 in SBP1, amino acid 57 was mutated from a cysteine (TGC) to a glycine (GGC) and expressed in mammalian cell lines. The obtained results indicate that cysteine 57 is a likely critical amino acid for both its interaction with GPx1 and its biological functions.

## 2. Results

### 2.1. SBP1^GLY^ Was Impaired in Its Ability to Protect HCT116 Cells from Selenite-Induced Toxicity

**Figure 1:**
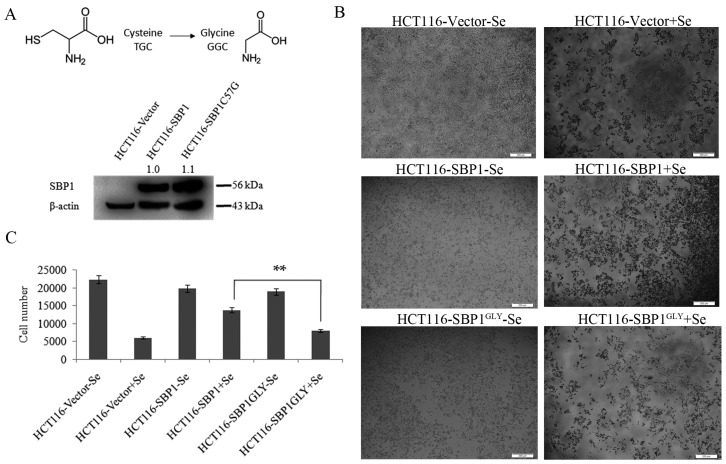
SBP1^GLY^ was impaired in its ability to protect HCT116 cells from selenite-induced toxicity. (**A**) Codon 57 of SBP1 was changed from a cysteine to a glycine and expression constructs representing the native and mutated SBP1 were introduced into HCT116 cells that do not produce detectable levels of SBP1. SBP1 signals were quantified and normalized to β-actin. Relative intensities were indicated; (**B**) HCT116 cells transfected with vector, pIRES2-SBP1, or pIRES2-SBP1^GLY^ were treated with 10 µM sodium selenite for 48 h and then photoed under microscope. Scale bar = 200 µm; and (**C**) cell number was quantified using ImageJSoftware. Results were analyzed with Student’s *t*-test and shown as mean ± SD. ** *p* < 0.01.

In order to investigate the biological consequences of the cysteine residue at codon 57 of SBP1, that amino acid was changed to a glycine by site directed mutagenesis (referred to a SBP1^GLY^) and expression constructs representing the native and mutated SBP1 were introduced into HCT116 cells that do not produce detectable levels of SBP1 (Figure 1A). PlasmidspIRES2-SBP1, pIRES2-SBP1^GLY^, or the empty vector pIRES2 were independently transfected into HCT116 cell sand the levels of SBP1 were determined by immunoblot analysis. As previously reported and shown in Figure 1A, SBP1 was essentially undetectable in HCT116, while very similar levels of the native and mutated SBP1 were achieved following transfection.

One of the consequences of SBP1 expression is the reduced sensitivity to the cytotoxic effects of sodium selenite [14]. In order to determine whether the cysteine residue located at position 57 was required for this protection, HCT116 cells expressing either the native or mutant SBP1 were exposed to 10 µM sodium selenite for 48 h and the number of surviving cells were quantified (Figure 1B,C). As seen in the figure, expression of SBP1 significantly protected HCT116 cells from selenium toxicity (38.2% survival *vs.* 73.2%). However, the expression of a similar level of SBP1^GLY^ was much less effective in protecting the cells from selenite toxicity as compared to SBP1 with a cysteine at position 57 (Figure 1B,C). 

### 2.2. The Cytosolic Half-Life of SBP1 Is Affected by the Amino Acid at Position 57

**Figure 2:**
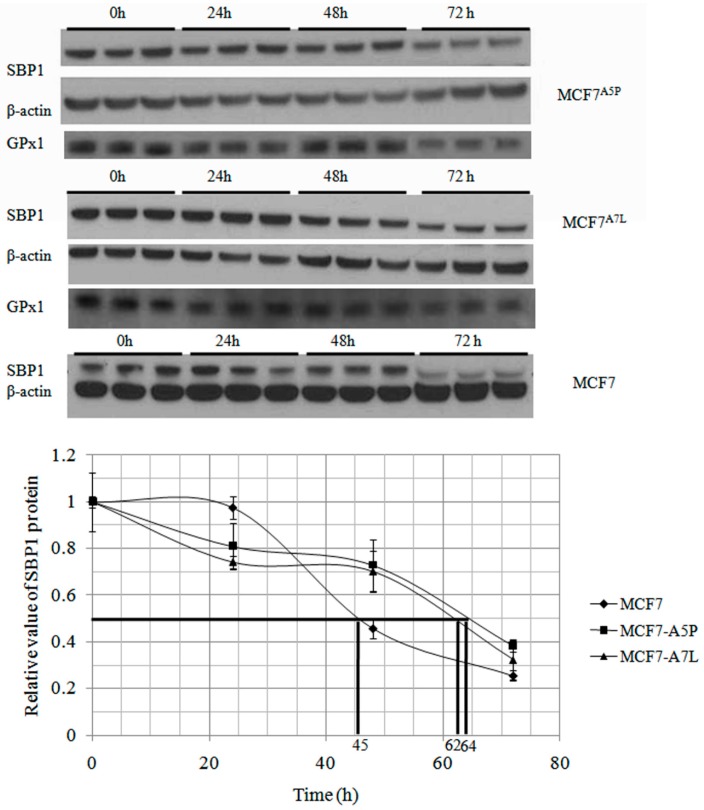
GPx1 extends endogenous SBP1 protein half-life. After treated with 100 µg/mL cycloheximide for 24, 48, and 72 h, different MCF7 derivative cells were harvested and protein levels of SBP1 were determined by immunoblot analysis. The signals were quantified by densitometry using ImageJ Software and normalized to β-actin. The determined half-life of SBP1 was 45, 64, and 62 h in MCF7, MCF7^A5P^, and MCF7^A7L^ cells, respectively. The differences between MCF7 and MCF7^A5P^, MCF7^A7L^ cells are statistically significant (*p* < 0.05).

**Figure 3:**
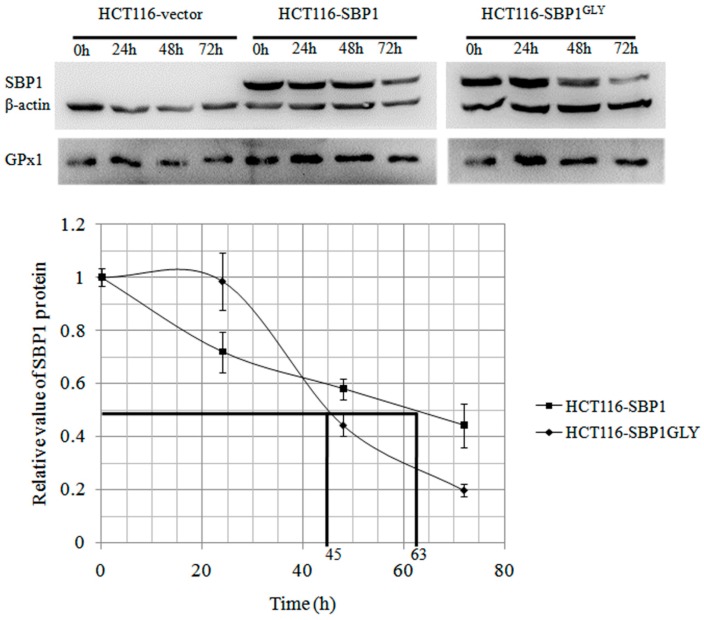
SBP1^GLY^ has a shorter protein half-life than SBP1. HCT116 cells transfected with vector, pIRES2-SBP1, or pIRES2-SBP1^GLY^ were treated with 100 µg/mL cycloheximide for 24, 48 and 72 h and then protein levels of SBP1 were determined by immunoblot analysis. The signals were quantified by densitometry using ImageJ Software and normalized to β-actin. The determined half-life of SBP1 and SBP1^GLY^ were 63 and 45 h in HCT116 cells which has a statistically significant difference (*p* < 0.05).

SBP1 has been shown to physically interact with the GPx1 selenoprotein [5] and consistent with this interaction, there have been several reports of reduced GPx1 enzyme activity when SBP1 is expressed, indicating a possible suppressive effect of that interaction [15]. The half-life of the native SBP1 and SBP1^GLY^ was determined as another means to assess the interaction between SBP1 and GPx1, assuming that the half-life would be altered by the interaction between these two proteins. This was done by determining the half-life of SBP1 in MCF7 cells that express undetectable levels of GPx1 or derivative clones that exclusively express different GPx1 alleles, MCF7^A5P^, and MCF7^A7L^ [16], suppressing new protein synthesis with cyclohexamide and measuring SBP1 levels by Western blotting at selected time intervals. The determined half-life of SBP1 was 45, 64, and 62 h in MCF7, MCF7^A5P^, and MCF7^A7L^ cells, respectively (Figure 2). The results established that the presence of GPx1 expressed from either of the tested alleles could extend endogenous SBP1 protein half-life to a similar extent. In order to determine whether the cysteine at position 57 affected SBP1 half-life, degradation of SBP1 and SBP1^GLY^ was determined in HCT116 that do not express detectable levels of endogenous SBP1 but do express significant levels of GPx1 [5]. SBP1 half-life was longer than SBP1^GLY^ in HCT116 cells, being 63 and 45 h, respectively (Figure 3).

### 2.3. Differential Affects of Wild Type SBP1 and SBP1^GLY^ on Signaling Pathways Relevant to Carcinogenesis

**Figure 4:**
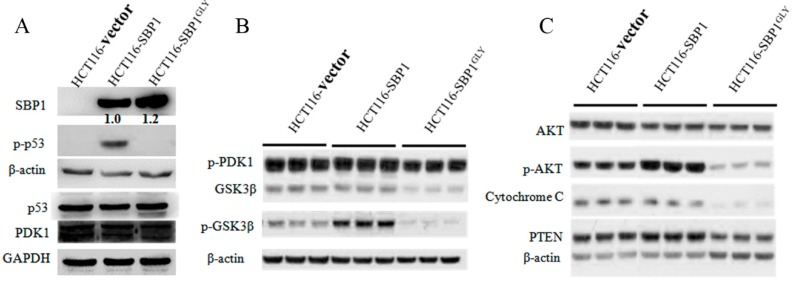
Differential effects of wild-type SBP1 and SBP1^GLY^ on signaling pathways relevant to carcinogenesis. (**A**–**C**) After 48 h of transfection of HCT116 cells with vector, pIRES2-SBP1, or pIRES2-SBP1C57G, protein levels including phospho-p53, p53, phospho-PDK1, PDK1, GSK3β, phospho-GSK3β, total AKT, phospho-AKT (serine 473), PTEN, and cytochrome C were examined by immunoblot analysis. SBP1 signals were quantified and normalized to β-actin. Relative intensities were indicated.

The expression of SBP1 is anticipated to impact signaling pathways associated with cancer and growth, perhaps by both direct and indirect mechanisms. This notion is supported by the recent assessment of changes in gene expression observed in HCT116 cells in an *in vivo* model where SBP1 was induced in mouse xenografts [17]. In this study, the expression of 132 genes were altered by SBP1 expression, many of the detected changes were in the transcription of genes involved in metabolism and growth control. In order to investigate the importance of cys^57^ for SBP1 function, representative proteins from several critical signaling pathways were assessed in SBP1 and SBP1^GLY^ expressing HCT116 cells. Specifically, the levels of phospho-p53, p53, phospho-PDK1, PDK1, GSK3β, phospho-GSK3β, total AKT, phospho-AKT (serine 473), PTEN, and cytochrome C were examined by immunoblot analysis 48 h after transfection (Figure 4). PTEN, p53 and cytochrome C are critical proteins in p53 signaling pathway while AKT, GSK3β and PDK1 are important molecules involved in AKT signaling pathway. These two different signaling pathways regulate the expression of a wide variety of genes involved in apoptosis, cell proliferation, cell cycle progression, differentiation and senescence, and play important roles in carcinogenesis. We have previously reported that the phosphorylation of p53 on serine15 is stimulated by the expression of SBP1 [18] and this is substantiated by the data shown in Figure 4A. In that figure, it is also evident that the mutation of cysteine 57 of SBP1 eliminated that effect. A similar result was obtained when phosphorylation of GSKβ was examined; expression of wild type SBP1 stimulated the phosphorylation of GSKβ while this was not observed when the mutant SBP1 was expressed (Figure 4B). While these data indicated the likely requirement for cysteine 57 for SBP1 function, a different result was obtained when the effects of SBP1 and SBP1^GLY^ were examined on the mitochondrial proteins cytochrome C, PTEN, and p-PDK1 (Figure 4C). While ectopic expression of SBP1 had a marginal effect if any on the levels of cytochrome C or the phosphorylation of PDK1, expression of SBP1^GLY^ lowered the levels of cytochrome C and reduced the levels of p-PDK1 well below that seen in control, vector-transfected HCT116 cells. Similarly, the levels of PTEN were reduced by the expression of SBP1^GLY^ but not the wild-type protein.

### 2.4. Expression of SBP1^GLY^ Induced Mitochondrial Damage in HCT116 Cells

**Figure 5:**
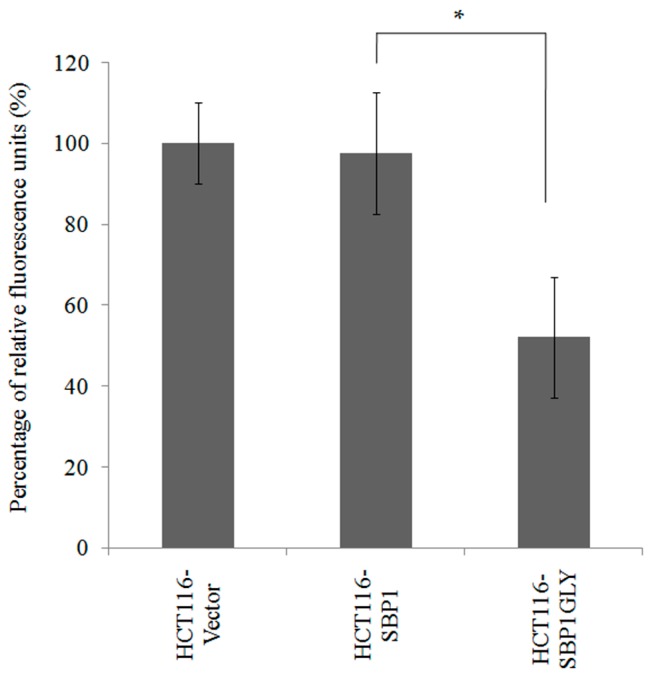
SBP1^GLY^ induced mitochondrial damage in HCT116 cells. Mitochondrial damage in HCT116 cells expressing either SBP1 and SBP1^GLY^, or the empty vector, were examined by using nonyl-acradine orange which assesses mitochondrial damage by staining cardiolipinin the inner mitochondrial membrane. Relative fluorescence units quantified using excitation and emission filters at 580 nm and 630 nm were reverse-related with cell mitochondrial damage. Results were analyzed with Student’s *t*-test and shown as mean ± SD. * *p* < 0.05.

Given the effects of SBP1^GLY^ on the mitochondrial proteins cytochrome C and p-PDK1 (Figure 4), it was assessed whether there was a differential effect of the two SBP1 proteins on mitochondrial integrity. This was done by transiently transfecting HCT116 cells with constructs that result in the expression of either SBP1 and SBP1^GLY^, or the empty vector and examining mitochondrial damage by using nonyl-acradine orange which assesses mitochondrial damage by staining cardiolipinin the inner mitochondrial membrane. Transient expression of SBP1 did not alter the degree of mitochondrial damage observed in recipient cells while there was significantly more damage in those cells transfected with SBP1^GLY^ (Figure 5).

## 3. Discussion

Using both experimental and computational approaches, Raucci *et al.* [4] concluded that the most likely binding site for selenium in SBP1 is the cysteine located at position 57. Based on those studies, cys^57^ was mutated to a glycine as a probe of the functional significance of that position. The selected approach utilized two previously characterized cell lines to achieve exclusive expression of either a mutated SBP1 or different alleles of GPx1, a previously reported SBP1 binding partner, following introduction of the appropriate expression construct. HCT116 cells do not express detectable SBP1 while MCF7 does not produce detectable GPx1.

As a first step in the analysis, HCT116 cells expressing either the wild-type or mutated SBP1 were examined for the sensitivity to selenite. It was previously shown that knocking down SBP1 sensitized cells to selenite toxicity due to altering the levels of extracellular glutathione, which accelerates uptake of selenium, leading to an increase in selenite-induced formation of reactive oxygen species (ROS) and cell death [14]. Consistent with this observation, expression of wild-type SBP1 was protective against selenite toxicity while, in contrast, the mutated SBP1 was not, despite both proteins being expressed at similar levels in the transfected cells (Figure 1). This observation is the first to provide an indication of the functional significance of cys^57^.

The impact of cys^57^ on the interaction between SBP1 and GPx1 was investigated in MCF7 cells that were either null for GPx1 or expressed different GPx1 alleles previously shown to differ in cancer risk and cellular location [15,16]. The presence of GPx1 altered the kinetics of turnover of SBP1, as shown in Figure 2, although GPx1 allelic specific differences were not observed. Given the demonstrated lack of differences between the effects of GPx1 alleles on SBP1 stability, the effect of the amino acid identity at position 57 of SBP1 was investigated in HCT116 cells expressing the wild-type or mutated SBP1 gene as well as endogenous GPx1. Given that the kinetics of turnover of SBP1 was similar in GPx1 null MCF7 cells (Figure 2) and when SBP1^GLY^ was being expressed in HCT116 cells (Figure 3), it is concluded that the cysteine at position 57 is likely to impact the interaction between SBP1 and GPx1.

The exclusive expression of the wild-type or mutated SBP1 in HCT116 cells also permitted the investigation as to whether cys^57^ affects signaling pathways implicated in carcinogenesis (Figure 4). The data obtained indicated two distinct consequences of the exchange of cys^57^ with glycine. One was the elimination of stimulatory effects observed when the wild type SBP1 was expressed, as was seen for the enhancement of the phosphorylation of p53, GSKβ, and AKT. The other was a reduction in the levels of cytochrome C, PTEN, and p-PDK1 below what was seen in the SBP1 null HCT116 cells. Inhibition of protein phosphorylation and these mitochondrial proteins may be due to the induction of mitochondrial damage and subsequent ATP reduction when the mutant SBP1 was expressed (Figure 5), although it is not possible to distinguish cause and effect between the loss of these proteins and mitochondrial integrity with these data.

Although the data presented herein cannot establish cys^57^ as being critical for selenium binding, it does establish this residue as one critical element for SBP1 function. Among those features impacted was its interaction with GPx1, a protein implicated in the risk of cancer and other diseases by human genetics [19,20] as well as the propagation of signal pathways relevant to cancer and other pathologies. Future studies should be directed towards obtaining direct experimental evidence of the mechanism by which selenium associates with SBP1 and better understanding the role of the selenium atom to SBP1 function.

## 4. Materials and Methods

### 4.1. Cell Culture and Reagents

The human colon carcinoma cell line HCT116 and human breast cancer cell lineMCF7 were obtained from ATCC (American Type Culture Collection, Manassas, VA, USA) and authenticated by Genetetica DNA Laboratories in 2013. Stable transfectants expressing the GPx1 allele containing five alanine repeats and a proline at position 198 (GPx1^A5P^) and the GPx1 allele containing seven alanine repeats and a leucine at position 198 (GPx1^A7L^) were generated previously [21,22]. All MCF7-derivative cells were maintained in DMEM medium (Life Technologies, Carlsbad, CA, USA). HCT116 cells were transiently transfected with plasmids pIRES2, pIRES2-SBP1, or pIRES2-SBP1^GLY^, respectively with the Lipofectamine 2000 transfection reagent (Life Technologies) following the manufacturer’s protocol and maintained in McCoy’s 5A medium (Mediatech, Manassas, VA, USA). All media were supplemented with 10% fetal bovine serum, 100 U/mL penicillin and 100 µg/mL streptomycin and cultures were maintained at 37 °C in a humidified incubator with 5% CO_2_. Sodium selenite (≥98%, Sigma-Aldrich, Shanghai, China) and cycloheximide (~98%, Sigma-Aldrich) were dissolved in phosphate buffered saline (PBS) at 1 mol/L and 100 mg/mL stock solution, respectively, and stored at −20 °C until used.

### 4.2. Plasmid Construction

Plasmids pIRES2 and pIRES2-SBP1 were previously generated [23] and the pIRES2-SBP1^GLY^ plasmid was made using the QuikChange II Site-Directed Mutagenesis Kit (Agilent Technologies, Santa Clara, CA, USA) following the manufacturer’s instruction with the forward primer 5′-CCCCAAGTCTCCCCAGTATGGCCAGGTCAT-3′ and the reverse primer 5′-ATGACCTGGCCATACTGGGGAGACTTGGGG-3′, and confirmed by sequencing.

### 4.3. Immunoblot Analysis

Harvested cells were lysed in 1× Cell Lysis Buffer (Roche, Shanghai, China) containing 1mM PMSF (Cell Signaling Technology, Danvers, MA, USA). After incubation on ice for 20 min, lysates were centrifuged at 10,000× *g* for 15 min at 4 °C. The protein concentration of the supernatant was quantified using the BCA protein assay kit (Beyotime Institute of Biotechnology, Shanghai, China). Sixty µg of each sample were applied to 12% SDS-PAGE gels and electroblotted onto PVDF membranes (Bio-Rad Laboratories, Hercules, CA, USA). The membrane was blocked with 5% non-fat milk in TBST (20 mM Tris, pH 7.6, 137 mM NaCl, 0.05% Tween-20) for 1 h at room temperature, and then probed with specific antibodies. Primary antibodies and dilutions including SBP1 at 1:1000 (mouse, MBL International, Woburn, MA, USA), GPx1 at 1:1000 (mouse, MBL International), β-actin at 1:2000 (mouse, Proteintech, Chicago, IL, USA), phospho-p53 at 1:1000 (rabbit, Proteintech), p53 at 1:1000 (rabbit, Proteintech), PDK1 at 1:1000 (rabbit, Proteintech), GAPDH at 1:1000 (rabbit, Proteintech), phospho-Akt (Ser473) at 1:1000 (rabbit, Cell Signaling Technology, Danvers, MA, USA), Akt at 1:1000 (rabbit, Cell Signaling Technology), PTEN at 1:1000 (rabbit, Cell Signaling Technology), Cytochrome C at 1:1000 (mouse, Santa Cruz, Santa Cruz, CA, USA), phospho-PDK1 at 1:1000 (rabbit, Cell Signaling Technology), GSK3β at 1:1000 (rabbit, Cell Signaling Technology), and phospho-GSK3β at 1:1000 (rabbit, Cell Signaling Technology) were incubated with the membrane at 4 °C overnight. The appropriate secondary antibody was applied (1:1000, rabbit or mouse) at room temperature for 1 h. The detected signals were visualized using BeyoECL Plus (Beyotime Institute of Biotechnology) and quantified by densitometry using ImageJ Software.

### 4.4. Measurement of Selenite-Induced Cytotoxicity

Equal amounts of HCT116 cells (1 × 10^4^/well, *n* = 5) were seeded in a 96-well plate (Corning Inc., Corning, NY, USA) and separately transfected with pIRES2, pIRES2-SBP1, or pIRES2-SBP1^GLY^. The medium was replaced with fresh culture medium containing 10 µM sodium selenite 24 h after transfection. Cells were quantified 48 h later by microphotography and cell numbers were quantified using ImageJ Software. The rate of cell death was calculated by dividing the number of cells obtained by the number of cells of the appropriate control group. The data presented is the result of three or more independent experiments.

### 4.5. Determination of the SBP1 Half-Life

Equal amounts of MCF7 derivative cells (MCF7, MCF7^A5P^, and MCF7^A7L^) and HCT116-derivative cells (transfected with pIRES2, pIRES2-SBP1, and pIRES2-SBP1^GLY^, separately) were seeded in a six-well plate, incubated for 24 h and then treated with 100 µg/mL cycloheximide for 24, 48, and 72 h. After the indicated treatment, the cells were harvested and the levels of SBP1 were determined by immunoblot analysis. The obtained signals were quantified by densitometry using ImageJ Software and normalized to β-actin.

### 4.6. Mitochondrial Damage Assay

HCT116 cells (10^4^/well, *n* = 5) were seeded in black, clear bottom 96-well assay plates (Corning Inc.) and transfected with either pIRES2, pIRES2-SBP1 or pIRES2-SBP1^GLY^. Mitochondrial damage was measured 48 h later using the Cell Mitochondrial Damage Assay kit (Genmed Scientifics Inc., Wilmington, DE, USA) following the manufacturer’s instructions. Relative fluorescence units were quantified using excitation and emission filters at 580 and 630 nm, respectively, and were inversely related with cellular mitochondrial damage.

### 4.7. Statistical Analysis

All values were represented by the mean ± S.D. of the indicated number of replicates. Statistical analyses of the data were performed using Student’s *t*-test to establish significance between data points. *p* < 0.05 were considered statistically significant.

## 5. Conclusions

Cysteine 57 in SBP1 is a critical determinant of SBP1 function, mutation at this point leads to high sensitivity to selenite cytotoxicity, shorter half-life of SBP1 protein, mitochondrial damage and differential effects on signaling pathways relevant to carcinogenesis. Therefore, Cysteine 57 plays a critical role in the biological functions of SBP1.

## References

[1] Chang P.W., Tsui S.K., Liew C., Lee C.C., Waye M.M., Fung K.P. (1997). Isolation, characterization, and chromosomal mapping of a novel cDNA clone encoding human selenium binding protein. J. Cell. Biochem..

[2] Chen G., Wang H., Miller C.T., Thomas D.G., Gharib T.G., Misek D.E., Giordano T.J., Orringer M.B., Hanash S.M., Beer D.G. (2004). Reduced selenium-binding protein 1 expression is associated with poor outcome in lung adenocarcinomas. J. Pathol..

[3] Huang C., Ding G., Gu C., Zhou J., Kuang M., Ji Y., He Y., Kondo T., Fan J. (2012). Decreased selenium-binding protein 1 enhances glutathione peroxidase 1 activity and downregulates HIF-1α to promote hepatocellular carcinoma invasiveness. Clin. Cancer Res..

[4] Raucci R., Colonna G., Guerriero E., Capone F., Accardo M., Castello G., Costantini S. (2011). Structural and functional studies of the human selenium binding protein-1 and its involvement in hepatocellular carcinoma. Biochim. Biophys. Acta.

[5] Fang W., Goldberg M.L., Pohl N.M., Bi X., Tong C., Xiong B., Koh T.J., Diamond A.M., Yang W. (2010). Functional and physical interaction between the selenium-binding protein 1 (SBP1) and the glutathione peroxidase 1 selenoprotein. Carcinogenesis.

[6] Huang K.C., Park D.C., Ng S.K., Lee J.Y., Ni X., Ng W.C., Bandera C.A., Welch W.R., Berkowitz R.S., Mok S.C. (2006). Selenium binding protein 1 in ovarian cancer. Int. J. Cancer.

[7] Kim H., Kang H.J., You K.T., Kim S.H., Lee K.Y., Kim T.I., Kim C., Song S.Y., Kim H.J., Lee C. (2006). Suppression of human selenium-binding protein 1 is a late event in colorectal carcinogenesis and is associated with poor survival. Proteomics.

[8] Li T., Yang W., Li M., Byun D.S., Tong C., Nasser S., Zhuang M., Arango D., Mariadason J.M., Augenlicht L.H. (2008). Expression of selenium-binding protein 1 characterizes intestinal cell maturation and predicts survival for patients with colorectal cancer. Mol. Nutr. Food Res..

[9] Xia Y.J., Ma Y.Y., He X.J., Wang H.J., Ye Z.Y., Tao H.Q. (2011). Suppression of selenium-binding protein 1 in gastric cancer is associated with poor survival. Hum. Pathol..

[10] Zhang J., Dong W.G., Lin J. (2011). Reduced selenium-binding protein 1 is associated with poor survival rate in gastric carcinoma. Med. Oncol..

[11] Jeong J.Y., Wang Y., Sytkowski A.J. (2009). Human selenium binding protein-1 (hSP56) interacts with VDU1 in a selenium-dependent manner. Biochem. Biophys. Res. Commun..

[12] Schild F., Kieffer-Jaquinod S., Palencia A., Cobessi D., Sarret G., Zubieta C., Jourdain A., Dumas R., Forge V., Testemale D. (2014). Biochemical and biophysical characterization of the selenium-binding and reducing site in *Arabidopsis thaliana* homologue to mammals selenium-binding protein 1. J. Biol. Chem..

[13] Porat A., Sagiv Y., Elazar Z. (2000). A 56-kDa selenium-binding protein participates in intra-Golgi protein transport. J. Biol. Chem..

[14] Wang Y., Fang W., Huang Y., Hu F., Ying Q., Yang W., Xiong B. (2015). Reduction of selenium-binding protein 1 sensitizes cancer cells to selenite via elevating extracellular glutathione: A novel mechanism of cancer-specific cytotoxicity of selenite. Free Radic. Biol. Med..

[15] Ansong E., Yang W., Diamond A.M. (2014). Molecular cross-talk between members of distinct families of selenium containing proteins. Mol. Nutr. Food Res..

[16] Bera S., Weinberg F., Ekoue D.N., Ansenberger-Fricano K., Mao M., Bonini M.G., Diamond A.M. (2014). Natural allelic variations in glutathione peroxidase-1 affect its subcellular localization and function. Cancer Res..

[17] Ying Q., Ansong E., Diamond A.M., Lu Z., Yang W., Bie X. (2015). Quantitative proteomic analysis reveals that anti-cancer effects of selenium-binding protein 1 *in vivo* are associated with metabolic pathways. PLoS ONE.

[18] Ansong E., Ying Q., Ekoue D.N., Deaton R., Hall A.R., Kajdacsy-Balla A., Yang W., Gann P.H., Diamond A.M. (2015). Evidence that selenium binding protein 1 is a tumor suppressor in prostate cancer. PLoS ONE.

[19] Zhuo P., Diamond A.M. (2009). Molecular mechanisms by which selenoproteins affect cancer risk and progression. Biochim. Biophys. Acta.

[20] Rayman M.P. (2009). Selenoproteins and human health: Insights from epidemiological data. Biochim. Biophys. Acta.

[21] Zhuo P., Goldberg M., Herman L., Lee B.S., Wang H., Brown R.L., Foster C.B., Peters U., Diamond A.M. (2009). Molecular consequences of genetic variations in the glutathione peroxidase 1 selenoenzyme. Cancer Res..

[22] Hu Y.J., Diamond A.M. (2003). Role of glutathione peroxidase 1 in breast cancer: Loss of heterozygosity and allelic differences in the response to selenium. Cancer Res..

[23] Pohl N.M., Tong C., Fang W., Bi X., Li T., Yang W. (2009). Transcriptional regulation and biological functions of selenium-binding protein 1 in colorectal cancer *in vitro* and in nude mouse xenografts. PLoS ONE.

